# Influence of Preclinical Training on Root Canal Treatment Technical Quality and Confidence Level of Undergraduate Dental Students

**DOI:** 10.1155/2021/9920280

**Published:** 2021-05-13

**Authors:** Reem M. Barakat, Manal Matoug-Elwerfelli, Rahaf A. Almohareb, Hanan A. Balto

**Affiliations:** ^1^Clinical Dental Sciences Department, College of Dentistry, Princess Nourah Bint Abdulrahman University, Riyadh, Saudi Arabia; ^2^Department of Restorative Dental Sciences, College of Dentistry, King Saud University, Riyadh, Saudi Arabia

## Abstract

**Objectives:**

The aim of this study was to investigate the influence of exposure to additional preclinical endodontic training on undergraduate students' technical quality of root canal treatment and overall confidence levels in endodontics.

**Methods:**

Technical quality of root canal treatment performed clinically by fifth-year undergraduate students was evaluated and divided into two groups: Group 1, teeth treated by students who had attended both a preclinical endodontic block course and an elective preclinical course. Group 2: teeth treated by students who had not attended the elective preclinical course. All students were also invited to participate in a survey to rate their undergraduate endodontic training and confidence levels performing endodontic treatment. Statistical analysis of data was performed using Person chi-square test, Fisher Freeman Halton exact test, and *t*-test. A *p*-value <0.05 was considered statistically significant.

**Results:**

There was no significant difference between the two groups in overall obturation quality (*p*=0.619). However, more teeth treated by attendees were of adequate obturation length (*p*=0.015) and lacked procedural errors (*p*=0.004). Significantly more elective course attendees rated their undergraduate endodontic training as adequate (*p*=0.002), but there was no significant difference in the level of confidence between the attendees and the non-attendees.

**Conclusion:**

Within the limitations of this study, additional preclinical training showed minimal effect on overall quality of root canal treatment performed clinically by undergraduate students and did not enhance their confidence levels; however, it was associated with more satisfaction with their undergraduate endodontic education.

## 1. Introduction

More than any other subject in the dental undergraduate curriculum, teaching endodontics has challenged those involved in delivering the required knowledge and skills [1]. Most students view this discipline as complex and difficult to master, which in turn reflects on how confident they feel when it comes to performing root canal treatment, especially on molars [2]. This has been echoed by the high frequency of inadequate root canal fillings and procedural errors [3, 4].

The desired outcome of an endodontic undergraduate curriculum is for graduating students to achieve clinical competence in performing root canal treatment of uncomplicated single-rooted and multi-rooted teeth [5]. While expert guidelines have specified the scope of endodontic education [6], preclinical and clinical teaching methodologies, including the minimum number of cases required to achieve competency, have remained vague [7]. Limited preclinical and clinical training time was cited as a major roadblock to attaining competency and thus achieving self-confidence in performing root canal treatment [1, 2, 8, 9]. Students have allocated special importance to preclinical training in endodontics in helping them acquire the necessary skills [2].

Previous studies have examined the effect of certain modifications in preclinical endodontic teaching, for example, training on artificial instead of natural teeth and preclinical exposure to the apex locator, by evaluating the technical quality of root canal treatment performed by the undergraduate students in a prospective manner [10, 11]. Little is known however about the influence of exposure to additional preclinical endodontic training on improving clinical skills and students' confidence levels.

The aim of the present study was to investigate the influence of extending preclinical endodontic training through an elective five-week preclinical course, on undergraduate students' confidence levels and the technical quality of root canal treatment. The alternative hypothesis was that students exposed to additional preclinical training would demonstrate higher confidence levels and improved endodontic clinical performance.

## 2. Materials and Methods

This prospective cohort study involved all registered fifth-year (final) undergraduate students (*n* = 46) at Princess Nourah Bint Abdulrahman University (PNU), College of Dentistry, during the academic year 2019–2020. Half of these student (*n* = 23) showed interest and registered to attend the elective endodontic preclinical course. The study was exempt from ethical approval by the institutional review board at PNU (IRB#19–0165).

Undergraduate endodontic teaching at PNU, College of Dentistry, starts with a preclinical block course in the third year composed of a theoretical component and hands-on preclinical sessions that run for seven weeks, culminating in a total of ninety contact hours. All preclinical training takes place in a simulated setting, on mannequin heads, on which students are expected to complete root canal treatment for plastic and natural anterior and posterior teeth (three anteriors, three premolars, and three molars), in addition to performing extra access cavity preparation training on six teeth and a retreatment on a premolar.

Upon passing this preclinical course, students can commence performing non-surgical root canal treatment on patients under close supervision as part of stream comprehensive clinical courses.

An elective advanced preclinical endodontic block course is also offered in the fourth year. This course runs for five weeks (a total of forty-five contact hours) and offers advanced theoretical knowledge along with simulated preclinical training sessions focused on molar teeth. During this course, students are required to complete root canal treatment on two premolars and three molars.

## 3. Case Selection

All completed non-surgical root canal treatment cases performed by the fifth-year students during the academic year were evaluated. Students performed root canal treatment at the College Of Dentistry's dental clinics under the supervision of endodontic specialists, with an average student to staff ratio of 1 : 7. An aseptic technique with rubber dam isolation was applied in all cases. Working length was established using electronic apex locator and periapical radiographs. Canal cleaning and shaping was done using either manual 0.02 mm taper stainless-steel k-files (Medin, A.S. Czech Republic) with step-back technique or rotary instrumentation using nickel-titanium Protaper Universal files (Dentsply Maillefer, Ballaigues, Switzerland). Canals were irrigated with sodium hypochlorite 2.25% for chemical disinfection, dried with paper points, and then filled with gutta-percha cones and AH plus sealer (Dentsply-Sirona, United States) using a lateral condensation technique. On completion, coronal restoration was performed, and a postoperative periapical radiograph was exposed using the paralleling technique.

The academic dental software system, AxiUm (Exan, BC, Canada), employed at the dental clinics, was used to specify all non-surgical root canal treatment cases completed by fifth-year students from the start of the academic year on the first of September 2019 until work at the dental clinics was abruptly terminated on the eighth of March 2020 due to the COVID 19 pandemic. Retreatment cases were excluded. During evaluation, records with poor quality or missing periapical radiographs were also excluded.

The cases were divided into two groups: Group 1 (attendees), teeth treated by students who had attended both the preclinical endodontic block course in the third year and the elective preclinical course in the fourth year. Group 2 (non-attendees), teeth treated by students who had not attended the elective preclinical course.

## 4. Assessment of Clinical Performance

### 4.1. Root Canal Treatment Quality and Procedural Errors

Clinical performance of the students was assessed by evaluating the technical quality of the performed root canal treatment and the presence of procedural errors. Technical quality was determined based on criteria for adequate root canal filling: distance between the end of the root canal filling and the radiographic apex, filling density, and the detection of procedural errors, which had been adopted by Barriesh-Nusaair et al. [4] and Balto et al. [3], and is summarized in Table 1. The tooth was considered as one unit, scored according to the presence of errors, and/or defects in obturation length and density in any of its canals, for clinical failure of one root will eventually lead to failure of the tooth [3].

Number of cases performed by each student was also recorded. Two endodontists with a minimum experience of three years, blinded to the treating students, evaluated independently the technical quality of canal obturations, and presence of procedural errors by studying the preoperative, working length, and postoperative radiographs. Mesial and distal angulated radiographs were included for multi-rooted teeth. Digital periapical radiographic images were viewed, and analyzed using Mipax (Microtek, Taiwan). All radiographs were examined at 1.5 magnification on the same 21-inch LCD monitor resolution (1920 × 1200 at 60 Hz) in a darkened room, and the same ambient conditions were sustained during all the radiographic evaluation. Each original digital image was manipulated by the investigator to enhance the contrast and brightness of the image to give the subjectively clearest image of the root canal and radiographic apex as recommended by Akdeniz and Soğur [12].

Prior to the actual study, intra- and inter-examiner reliability were determined by evaluating eighteen endodontically treated teeth randomly selected from AxiUm records; these were not included in the study. These cases were evaluated twice by the same examiners, four weeks apart. Inter- and intra-examiner agreement were measured by Cohen's kappa (*k*). Values for inter-examiner agreement for obturation length, density, and procedural errors were *k* = 0.702, *k* = 0.667, and *k* = 0.824 respectively, while intra-examiner reliability ranged within (*k* = 0.77, −*k* = 1). This indicated good to excellent agreement [13]. Therefore, as reported by previous studies [10, 14, 15], it seemed acceptable to randomly allocate the sample radiographs equally between both endodontists.

### 4.2. Undergraduate Students' Confidence Survey

All students completing the five-year dental program in 2019–2020 were invited to participate in an online questionnaire survey at the beginning of their internship year in July 2020. Prior to completing the questionnaire, the students were made aware of the objectives of the survey and how the results will be used. They were informed that participation is voluntary, their answers will remain entirely anonymous, and they can withdraw at any time during or after completing the questionnaire.

The questionnaire adapted from Davey et al. [9] and Murray and Chandler [16] contained thirteen multiple-choice format questions and three open-ended questions. The questionnaire indicated students' attendance of the endodontic elective course, assessed their experiences, and perceived competence performing root canal treatments, along with their self-rated levels of the confidence in carrying out various endodontic tasks and their views on their undergraduate endodontic training. Participants were asked to classify their perceived level of confidence over a five-point scale: very confident, confident, neutral, low confidence, or extremely low confidence.

### 4.3. Statistical Analysis

Statistical analysis was performed using the Statistical Package for Social Sciences software (SPSS version 27.0 IBM Inc., Chicago, IL, USA). Descriptive data analysis was carried out and Person chi-square test and Fisher Freeman Halton exact test were conducted to analyze the categorical data: root canal filling quality, confidence level, and satisfaction. Independent samples *t*-test was used to compare the number of teeth completed students in each group. A *p*-value <0.05 was considered statistically significant.

## 5. Results

### 5.1. Assessment of Endodontic Technical Quality and Procedural Errors

One hundred and ninety teeth were evaluated through the AxiUm software system. Thirty-one teeth were excluded either due to missing or unclear radiographs, or records revealing that the treatment was incomplete. Of the remaining one hundred and fifty-nine treated teeth, the majority (95.6%) were molars (*n* = 69) and premolars (*n* = 83); the obturations were regarded as adequate quality in 60.38% (*n* = 96) of the cases. Procedural errors were recorded in thirty teeth (18.8%), of which apical perforation and instrument separation were the most recorded: 50% and 26.6%, respectively.

On assessing the obturation quality, 63.5% (*n* = 40) of the teeth treated by elective course attendees, and 58.33 % (*n* = 56) of non-attendee cases were regarded as adequate quality.  Table 2 shows distribution of obturation quality according to tooth type among students in both groups. Although significantly more teeth treated by attendees were of adequate obturation length (*p*=0.015) and lacked procedural errors (*p*=0.004), there was no significant difference between the two groups in overall obturation quality (*p*=0.619). The mean number of teeth treated by students who had attended the elective course (2.74) was significantly lower than that of non-attendees (4.17) (*p*=0.012).

### 5.2. Undergraduate Students' Perception and Confidence Survey

The questionnaire response rate was very high 97.8%. All students who had attended the elective course participated. When asked about their reason for attending the course, twelve students (52.1%) stated the desire to improve their hand skills and performance, while the remaining eleven students (47.9%) were motivated by their interest in endodontics.

**Figure 1:**
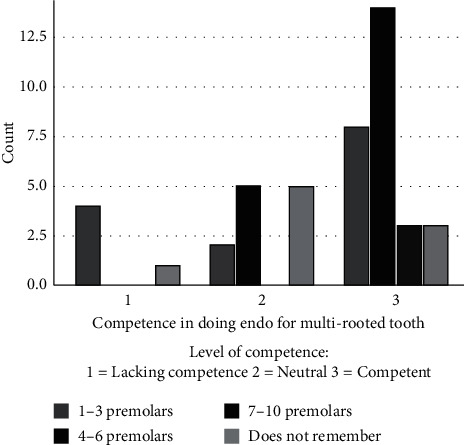
Number of treated premolars and perceived level of competence.

In regard to students' confidence level in performing different stages of root canal treatment, there was no significant difference in the level of confidence between the attendees and the non-attendees (Table 3). Although more students who attended the elective course perceived themselves competent in treating multi-rooted teeth (Table 4), this was not statistically significant (*p*=0.069). Attending the elective course was also not associated with a significant increase in the perception of competence in treating single-rooted teeth (*p*=1.00). However, the number of treated premolars was significantly associated with a perception of competence in treating multi-rooted teeth (*p*=0.043) (Figure 1).

On asking students about their perceived quality of undergraduate education, elective course attendees were more likely to describe all aspects of their undergraduate endodontic training as adequate (Table 5). This proved significant concerning the overall undergraduate training and theory content (*p*=0.002), (*p*=0.006), respectively. Another element associated with having an adequate opinion of undergraduate endodontic training was the student's perception of being competent in performing endodontic treatment on both single and multi-rooted teeth (*p*=0.021) (*p*=0.038).

In regard to the open-ended questions targeting student opinion and feedback on their undergraduate endodontic training, only nineteen students (42%) completed these questions. Students clearly expressed the desire to increase clinical and preclinical endodontic training hours, with a special focus on the use of rotary instrumentation and managing endodontic procedural errors.

## 6. Discussion

The issue of time in education is a matter of concern as it incurs greater costs and effort [17]. The undergraduate students in this study were supervised and taught by the same instructors; in relatively equal clinical training conditions only half of them had undergone the extended preclinical training. This permitted investigating the effect of extending preclinical training on undergraduate students' confidence levels and clinical performance of root canal treatment [18, 19].

The present findings showed no significant difference between the teeth performed by students who had attended the elective course and those who had not, as far as the overall technical quality of root canal treatment is concerned. At first glance, it appears that preclinical training time did not influence the undergraduate students' clinical performance of root canal treatment. This can be explained by what Chambers [20] described as the “learning curve” relationship between practice and competence, where a certain point is reached, beyond which extra practice no longer lends to the acquisition of skill. A study also looking into the acquisition of medical clinical skills through practice concluded that while the amount of practice time played a role in achieving an “initial mastery” of basic skills, progress beyond that was not achieved through repetition, but by performing tasks that specifically target the areas of performance deficiency [21].

However, students who did not attend the elective preclinical course performed significantly more procedural errors than those who attended. Overall, the frequency of procedural errors (18.8%) was fairly low which is in accordance with other studies [14, 15, 22, 23].

Adequate root canal treatment was seen in 60.3% of the cases performed by the undergraduate students, in spite of the fact that the majority of the treated teeth were posterior teeth (premolars and molars) which are usually associated with lower frequencies of adequate treatment when performed by undergraduate dental students (49.5% in premolars and 26.3% in molars) [24]. An explanation may have been that the students were constantly supervised by endodontists with a low student to instructor ratio [7, 15]. Another explanation may be the exclusion of root canal filling taper as an evaluation criterion of root canal filling quality in this study [19, 25]. Studies considering taper had reported lower frequencies of adequate root canal treatment [3, 4, 23]. Taper is a subjective matter that cannot be adequately reflected in a two-dimensional radiograph; moreover, it may be influenced by the canal's original anatomy giving the impression of a large taper irrespective of actual instrumentation [26]. Accordingly, it was excluded as an evaluation criterion in this study [19, 25].

The mean number of teeth completed by non-attendees was significantly higher compared to attendees. One possible explanation is that non-attendees sought out more opportunities to practice through clinical experience. Other factors that could lend an explanation to this result are the fact that the academic year was cut short due to the COVID-19 pandemic, and that retreatment cases performed by the students were not accounted for in the study [27].

Although more attendees (78.3%) perceived themselves competent in treating multi-rooted teeth compared to non-attendees 45.4%, survey results showed no significant difference between the two groups in confidence levels whether in performing a particular stage of endodontic treatment or in their self-perception of competence in treating single or multi-rooted teeth. These findings are in line with other studies exploring what was termed self-efficacy, a combination of competence and confidence-of students [25, 27], which found no difference between students who attended different theoretical and clinical modules. Confidence levels in this study were also in close proximity to those reported by similar studies despite the difficulty in drawing conclusions from other studies due to variations in methodologies [9, 16, 28, 29].

The difference between attendees and non-attendees lies in their satisfaction with the quality of their endodontic undergraduate education. When asked about their perception of the overall training quality of endodontic education, 87% (*n* = 20) of elective course attendees reported adequate training, in contrast to only 45.4% (*n* = 10) of non-attendees.

Students attribute great importance to preclinical training in helping them acquire the necessary skills in root canal treatment [2], which would explain this significant difference in the level of satisfaction. Sukotjo et al. [30] found that shortening preclinical hours in the undergraduate dental curriculum affected student anxiety and stress levels especially those measured prior to entering the clinics. The replies to the open-ended questions at the end of the questionnaire echoed this desire for more training time (clinical and preclinical), similar to what has been reported by previous studies when sounding out student perspectives on their undergraduate education [1, 2].

A recent study by Baaij and Özok [25] concluded that undergraduate students' feelings of competence and confidence were influenced by clinical experiences in endodontics, specifically successful ones. In accordance with this, the findings of the present study show that students' self-perception of competence was associated with the number of teeth they reportedly treated, in particular premolars. A reason for that maybe is students were required to pass an endodontic competency assessment, which involved completing root canal treatment on a two-canal upper premolar.

Certain limitations to this study should be kept in mind. The sample size was small as students from only one academic year were included, because the course was unavailable to students from the previous years. Student enrollment in the elective course was voluntary which would imply a risk of self-selection bias. The study investigated the attendees' reasons for taking the course: either a desire to increase skill or having an interest in endodontics. However, the reason why non-attendees did not choose to take the elective course was not explored. This could have shed more light on specific characteristics of that group.

Students' innate cognitive, perceptual, and psychomotor abilities related to acquiring competence in psychomotor skills were also not investigated [31]. Studies have shown that these abilities may play a role during the initial stages of skill acquisition and their influence tends to diminish with increased training [31]. However, innate spatial abilities remained of influence regardless of practice, for tasks of an important spatial nature, which could very well apply to procedures in root canal treatment [32].

Case difficulty which has a major part to play in undergraduate root canal treatment performance [22] was not taken into consideration in this study. It was assumed that endodontists supervising the students assess the case and refer moderate to high difficulty cases to post-graduate students or endodontic specialists within the Dental College's clinics.

Although the overall technical quality of root canal treatment performed by undergraduate students and their confidence levels were not affected by their preclinical training time, students who attended the elective course performed significantly fewer procedural errors and were more satisfied with their undergraduate endodontic education.

In spite of students' perspective regarding lack of training time, more focus should be placed on how preclinical endodontics is taught, how to facilitate deliberate practice, and not how much, i.e., the quality not the quantity [30]. Further research is required on pinpointing the challenges of acquiring of psychomotor skills required in root canal treatment and introducing novel teaching strategies targeting these unique skills. That said, it would also be interesting to further extend the preclinical course and observe whether the learning curve modifies.

## 7. Conclusions

Within the limitations of this study, additional preclinical training showed minimal effect on overall quality of root canal treatment performed clinically by undergraduate students and did not enhance their confidence levels. However, students who attended additional preclinical training performed significantly fewer procedural errors and were more satisfied with their undergraduate endodontic education. Further research is required on how to improve the training of undergraduate dental students in the field of endodontics.

## Figures and Tables

**Table 1:** Criteria for detection of procedural errors and technical quality of root canal treatment.

Criteria for detection of procedural errors		
Criteria		Definition
Ledge formation		The obturation was at least 1 mm shorter than the working length and deviated from the original canal shape in teeth where root canal curvature occurred
Apical transportation		In the apical third, the obturation was located on the outside curve of the canal
Apical perforation		The apical termination of the filled canal was different from the original canal terminus or when the obturation extruded through the apical foramen
Root perforation		Extrusion of canal obturation in any other area of a root except the furcation area and the inner wall of the root
Strip perforation		Extrusion of canal obturation in the lateral (inner) wall of the root canal
Presence of fractured instrument		A fractured instrument was detected inside a root canal or with its tip extending into the periapical area
Furcation perforation		Extrusion of filling material through the furcation area in multi-rooted teeth

Criteria for evaluation of technical quality of root canal treatment		
Length of root canal filling	Adequate	End of root canal filling is either at or ≤ 2 mm from radiographic apex
	Inadequate	Root canal filling is either too short (more than 2 mm from the radiographic apex) or extends beyond the radiographic apex
Density of root canal filling	Adequate	No voids are visible within the root canal filling or between the filling and the canal walls
	Inadequate	Voids are visible within the root canal filling or between the filling and the canal walls
Presence of procedural errors	Present	One or more procedural errors are detected
	Non present	No procedural errors detected

**Table 2:** Obturation quality of treated teeth according to tooth type among students who attended and did not attend the endodontic elective course.

Tooth type		Molars *N* = 69				Premolars *N* = 83				Anteriors *N* = 7			
Attend elective		Yes *N* = 27		No *N* = 42		Yes *N* = 35		No *N* = 48		Yes *N* = 1		No *N* = 6	
Technical quality of obturation		*N*	%	*N*	%	*N*	%	*N*	%	*N*	%	*N*	%
Obturation length	Adequate	26	96.29	25	59.52	33	94.28	43	89.58	1	100	4	66.66
	Overfill	0	0	8	19.04	1	2.85	2	4.16	0	0	2	33.33
	Short	1	3.70	6	14.28	1	2.85	1	2.08	0	0	0	0

Obturation density	Adequate	15	55.55	24	57.14	27	77.14	39	81.25	0	0	6	100
	Inadequate	12	44.44	15	35.71	8	22.85	8	16.66	1	100	0	0

Procedural errors	No errors	24	88.88	24	57.14	33	94.28	41	85.41	1	100	5	83.33
	Errors	3	11.11	18	42.85	2	5.71	7	14.58	0	0	1	16.66

Overall obturation quality	Adequate	14	51.85	15	35.71	26	74.28	37	77.08	0	0	4	66.66
	Inadequate	13	48.14	27	64.28	9	25.71	11	22.91	1	100	2	33.33

**Table 3:** Self-evaluated level of confidence in performing different stages of root canal treatment among elective course attendees (A) and non-attendees (NA).

Perceived level of confidence	Very confident		Confident		Neutral		Little confidence		Very little confidence		*p*-value
	A (%)	NA (%)	A (%)	NA (%)	A (%)	NA (%)	A (%)	NA (%)	A (%)	NA (%)	
Endodontic diagnosis	60.8	63.6	34.7	36.3	4.5	0	4.3	0	0	0	1.00
Patient referral for complicated cases	39.1	50	43.4	45.4	17.3	0	4.3	4.5	0	0	0.166
Providing adequate anesthesia	39.1	45.4	34.7	45.4	21.7	4.5	0	4.5	0	0	0.413
Rubber dam placement	60.8	63.6	34.7	31.8	0	4.5	4.3	0	0	0	1.00
Access cavity preparation	30.4	45.4	43.4	31.8	17.3	22.7	0	0	0	0	0.627
Finding all canals in multi-rooted teeth	0	4.5	47.8	36.3	34.7	22.7	4.3	36.3	0	0	0.301
Determining working length	30.4	27.2	65.2	54.5	4.5	18.1	17.3	0	0	0	0.423
Cleaning and shaping root canal system	26	50	56.5	39.1	17.3	9	0	0	0	0	0.275
Selecting proper irrigant and irrigation technique	43.4	45.4	43.4	45.4	13	9	8.6	0	0	0	1.00
Placing an inter-appointment dressing	34.7	54.5	52.1	22.7	8.6	13.6	0	9	0	0	0.242
Inter-appointment flare-ups management	4.3	18.1	39.1	30.4	39.1	27.2	4.3	22.7	0	0	0.489
Root canal system obturation	13	36.3	73.9	40.9	13	18.1	17.3	4.5	0	0	0.094
Taking radiographs	52.1	59	34.7	27.2	13	9	4.3	4.5	0	0	0.870
Interpreting radiographs	47.8	54.5	43.4	31.8	8.6	13.6	0	0	0	0	0.693
Giving postoperative instructions	52.1	72.7	43.4	13.6	4.3	13.6	0	0	0	0	0.066
Assessing quality of a root filling postoperatively	60.8	68.1	34.7	18.1	4.3	13.6	0	0	0	0	0.299
Determining correct recall period for patient	39.1	50	39.1	31.8	13	13.6	0	4.5	0	0	0.903
Performing non-surgical root canal retreatment	39.1	36.3	47.8	40.9	8.6	13.6	0	4.5	0	4.5	0.935

**Table 4:** Perceived competence in performing root canal treatment according to tooth type and elective course attendance.

Perceived competence in root canal treatment	Lacking				Neutral				Competent			
Tooth type	Single-rooted		Multi-rooted		Single-rooted		Multi-rooted		Single-rooted		Multi-rooted	
Attended elective	Yes	No	Yes	No	Yes	No	Yes	No	Yes	No	Yes	No
%	0	0	8.7	13.6	4.3	4.5	13	40.9	95.6	95.4	78.3	45.4

**Table 5:** Students' perception regarding the quality of their undergraduate endodontic education.

Perceived quality of undergraduate education		Attended elective endodontic course	
		Yes (*N* = 23) *N* (%)	No (*N* = 22) *N* (%)
Training time	Lacking	6 (26.1%)	10 (45.5%)
	Neutral	4 (17.4%)	4 (18.2%)
	Adequate	13 (56.5%)	8 (36.4%)

Lectures	Lacking	0	5 (22.7%)
	Neutral	1(4.3%)	4 (18.2%)
	Adequate	22 (95.7%)	13 (59.1%)

Lab	Lacking	6 (26.1%)	6 (27.3%)
	Neutral	1 (4.3%)	4 (18.2%)
	Adequate	16 (69.6%)	12 (54.5%)

Overall training	Lacking	3 (13%)	4 (18.2%)
	Neutral	0	8 (36.4%)
	Adequate	20 (87%)	10 (45.4%)

## Data Availability

The data that support the findings of this study are available from the corresponding author upon reasonable request.
